# Letter to the editor: Torpedo Maculopathy: A case series – insights into basic pathology

**DOI:** 10.1177/1120672120957595

**Published:** 2020-09-15

**Authors:** Diya Baker, Intesar Nur

**Affiliations:** 1 Sandwell and West Birmingham Hospitals NHS Trust, Birmingham, UK; 2 University of Birmingham College of Medical and Dental Sciences, Birmingham, UK

**Keywords:** Retinal Detachment, Retinal Pathology / Research, Congenital and Stationary Retinal Disease, Retinal Breaks

Dear Goncalo C Almeida,

This article was absorbing to read and it is fascinating to consider the breadth of mysteries within the macula that we have to elucidate. Upon reading your article, I had a number of comments with which I hoped to gain some clarity.

The study did not detail if there was accumulation of fluid throughout life in the patient cohort studied. Is there any data from your patient cohort regarding this?

Although this goes against the principles of old age leading to type 2, outer retinal cavitation present due to torpedo maculopathy, as stated by Wong et al^1^ there could be scope to look into this since despite this principle there is a patient of 37 years old that challenges this principle and is affirmed by the Mesnard et al. study which showed that type 2 is plausible even in young children.^2^

The question relating to accumulation of fluid throughout life may prove to be true as there has been a case by Su and Gurwood A where it has caused neurosensory retinal detachment.^3^ This proves the point on the matter that there may be fluid accumulation later in life. A study by Hamm et al. have found that abnormal choroid circulation could be a cause for it.^4^ They too were perplexed by the notion of the **cause, is it** due to a long term dysfunction or a primary change causing it? The paper did show that there is hypo-fluorescence with fundus auto-flourescence which atrophy or missing RPE as stated. As mentioned in your paper, is that these areas of atrophy give opportunity for fluid accumulation and this could be due to the changes in circulation around the choroid. One of the figures in their study goes to show less vessels in the sub retinal cleft but a higher choroidal vasculature which may be where the fluid could be accumulating from.

Contrastingly, a literature review conducted by Williams et al showed that **it** is more likely to be a congenital aetiology.^5^ This is due to the study showing that in the 77 cases majority were near the temporal macula and many were very young which is suggestive of an embryological foundation to which the disease is occuring. Long term follow up cases did not note any clinically significant changes suggesting that torpedo maculopathy is a progressive condition is less likely.

Instead, there may be multiple conditions people acquire throughout the ageing process which may be leading to the progressive accumulation of fluid rather than torpedo maculopathy.

A case study in India has shown multifocal central serous chorioretinopathy, the author clearly states this may not be linked to torpedo maculopathy.^6^ The serous fluid build up could be due to different causes and would need to be investigated further but it need not necessarily be the torpedo maculopathy that causes the progressive accumulation of fluid throughout life. Shields C et al has noted that there is a persistent defect in the RPE in the fetal temporal bulge.^7^ This gives more evidence towards a congenital potentially static view of torpedo maculopathy.
